# Nitriding Behaviour and Microstructure of High-Nitrogen Stainless Steel during Selective Laser Melting

**DOI:** 10.3390/ma16062505

**Published:** 2023-03-21

**Authors:** Xin Sun, Jianbiao Ren, Yachao Wang, Dingguo Zhao, Shuhuan Wang, Xiaojing Xiong, Jeremy Heng Rao

**Affiliations:** 1 School of Metallurgy and Energy, North China University of Science and Technology, Tangshan 063009, China; 2 Ji Hua Laboratory, Institute of Advanced Additive Manufacturing, Foshan 528200, China

**Keywords:** high-nitrogen stainless steel, selective laser melting, nitrogen content, microstructure, microhardness

## Abstract

High-nitrogen stainless steels are widely used due to their excellent comprehensive performance. In this study, the effects of process parameters (laser power, scanning speed, and cavity pressure) on the formation of high-nitrogen stainless steels were studied by using conventional selective laser melting and high-pressure selective laser melting (HPSLM). The nitrogen content, nitrogen emission, phase composition, microstructure, and microhardness of the high-nitrogen stainless steel samples obtained through selective laser melting (SLM) were analysed by using an oxygen/nitrogen/hydrogen analyser, X-ray diffraction, scanning electron microscopy, energy-dispersive X-ray spectroscopy, and electron backscatter diffraction. The results showed that the maximum nitrogen emission in the SLM sample was 0.175 wt.%, the emission rate reached up to 54.7%, and the maximum nitrogen content in the HPSLM sample was 1.07 wt.%. There was no significant difference between the phase peak positions of the SLM samples with different laser powers and the original powder. The main phase of the HPSLM sample changed at 0.3 MPa (from α-Fe to γ-Fe phase); the microstructure of the SLM sample was mainly composed of columnar and cellular crystals, and columnar crystal bands formed along the direction of heat flow. The HPSLM sample was mainly composed of equiaxed crystals with a grain size of 10–15 μm. At an energy density of 136 J/mm^3^, the microhardness and relative density reached their peak values of 409 HV and 98.85%, respectively.

## 1. Introduction

High-nitrogen stainless steels have excellent mechanical properties [1] and good corrosion resistance [2] and can improve the compatibility of stainless steels [3,4]; they are widely used in aviation [5], medicine [6], marine engineering [7], and petrochemical industries [8]. However, the solubility of nitrogen in austenitic molten steel is only approximately 0.4% at atmospheric pressure and approximately 0.08% in ferritic molten steel [9]. Common methods of the production of high-nitrogen stainless steel include [10] nitrogen pressure melting, powder metallurgy, surface nitriding, and high-temperature bulk nitriding [11,12,13].

Metal additive manufacturing technology is considered to be one of the major breakthroughs in the field of manufacturing of the last 30 years—it is even considered to promote the third industrial revolution [14]. Compared to traditional manufacturing methods, laser-based manufacturing is a widely used, noncontact, advanced manufacturing technique [15]. Selective laser melting (SLM) [16,17] is an important branch of additive manufacturing technology. The application of SLM to metal products has become a focus in the manufacturing industry in recent years, and the preparation of metal products through high-power laser irradiation of metal powders is one of the development trends. Kruth et al. [18] conducted a comprehensive study on the theory, process, and application of SLM technology. Mahyar Khorasani et al. [19] studied the effect of absorption ratio on meltpool features in the laser-based powder bed fusion of IN718 and showed the quality of the parts and transition from keyhole to conduction mode, emphasizing the importance of laser absorptivity. Kang et al. [20] extensively studied the microscopic mechanism of SLM. At present, materials produced by using SLM mainly include titanium-based alloys [21], nickel-based superalloys [22], iron-based alloys [23], and aluminum alloys [24]. Toschi et al. [25] observed that laser power affects the difference in the hardness between the top and bottom of large SLM specimens, laser power and scanning speed significantly affect the relative density, and the scanning rotation angle has no effect on the relative density of the sample. Tucho et al. [26] found that in the energy density range of 50–80 J/mm^3^, the porosity of SLM-formed 316 L stainless steels decreased exponentially with an increase in energy density, while the hardness increased linearly. Under the combination of a low scanning interval and high scanning speed, more compact printed parts can be prepared. Simmons et al. [27] determined the critical value of the scanning speed for 316 L stainless steels fabricated by performing SLM and analysed its effect on the porosity of printed parts. Nesma et al. [28] optimised the process parameters of SLM manufacturing for AlSi10Mg to achieve a printing density of 99.8%. However, the application of SLM technology to high-nitrogen stainless steels has rarely been reported. One possible reason is nitrogen escape in the large molten pool during the conventional smelting of high-nitrogen stainless steels and in the micro-molten pool formed during SLM preparation. The effects of the process parameters on the amount of nitrogen escaping and a solution to the nitrogen escape problem during SLM have not yet been reported. Therefore, a high-pressure SLM process is proposed in this study and the core feature of this method is the increase in nitrogen in the high-pressure atmosphere of molten steel, micro-pool nitriding mass transfer, and rapid solidification inhibition of nitrogen segregation.

In this study, the effects of process parameters on the nitrogen content, microstructure, and microhardness of high-nitrogen stainless steel samples obtained through SLM were investigated by using SLM and high-pressure selective laser melting (HPSLM) methods.

## 2. Materials and Methods

**Figure 1:**
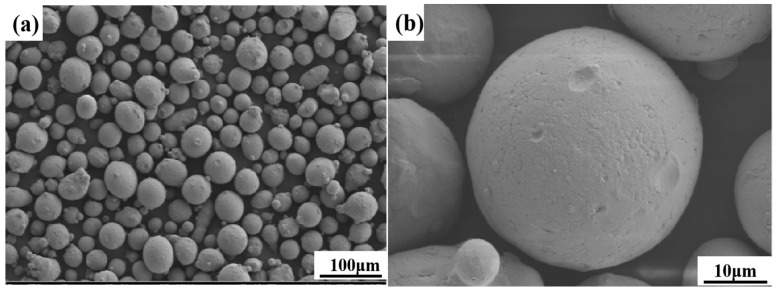
Microstructure of high-nitrogen stainless steel: (**a**) 100μm resolution; (**b**) 10μm resolution.

In this study, high-nitrogen stainless-steel metal powder was prepared by employing the gas atomisation method. The particle size of the powder was 15–53 μm, tap density was 4.9 g/cm^3^, and bulk density was 4.2 g/cm^3^. The morphology of the powder is shown in Figure 1. The powder particles are spherical, and some of the small particles are attached to the large ones in a satellite-like fashion. The chemical composition is presented in Table 1.

**Figure 2:**
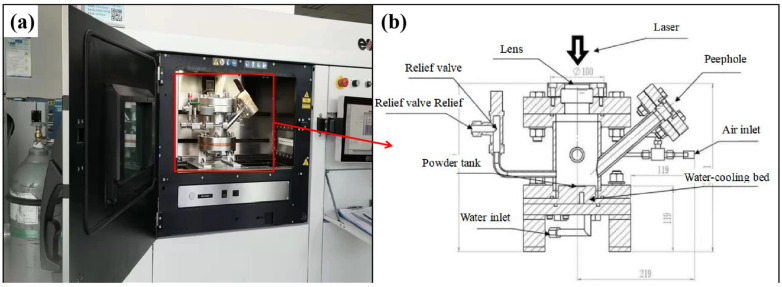
High-pressure melting chamber: (**a**) overall view of the SLM equipment; (**b**) profile view of high-pressure melting chamber.

SLM equipment (EOS M290, Germany) and an independently developed high-pressure melting chamber were used and the experimental setup is shown in Figure 2. The powder-laying groove was made of pure copper. Three parameters, namely, laser power (100–300 W), scanning speed (200–1000 mm/s), and cavity pressure (0.1–1.5 MPa), were considered as variables. The thickness of the scanning layer was set to 0.02 mm, and the scanning interval was set to 0.11 mm. The angle between the scanning lines in the Nth and N+1th layer was 67°. HPSLM was performed in a single layer.

The sample was separated from the substrate by using an electric-spark wire-cutting machine, the surface impurities were removed by grinding, and an ultrasonic cleaner was used to clean the surface. The nitrogen content in the formed sample was measured by using an oxygen/nitrogen/hydrogen tester (LECO, USA). The sample surface was polished to a mirror finish by employing 400#, 800#, 1000#, 1200#, 1500#, and 2000# abrasives.

X-ray diffraction (XRD; diffraction angle of 20–100°) was used to determine the phase composition. The grain boundary was corroded by using a corrosion solution (anhydrous copper sulfate:concentrated hydrochloric acid:distilled water = 4 g: 20 mL: 20 mL), and the microstructure was observed. Vickers hardness was used to determine the microhardness of samples with different energy densities. The hardness of the SLM and HPSLM high-nitrogen stainless steel samples was tested. The test direction was cross-measurement, and the interval between different test points was 0.2 mm. The average value of nine hardness measurement points was taken. The nitrogen content of the alloys produced by selective laser melting was measured by a LECO oxygen-nitrogen-hydrogen gas analyzer. The calculation of multicomponent phase diagrams was performed using the FactPS and FTOxCN databases in the FactSage software package.

## 3. Results

### 3.1. Effects of Process Parameters and Cavity Pressure on Nitrogen Contents

**Figure 3:**
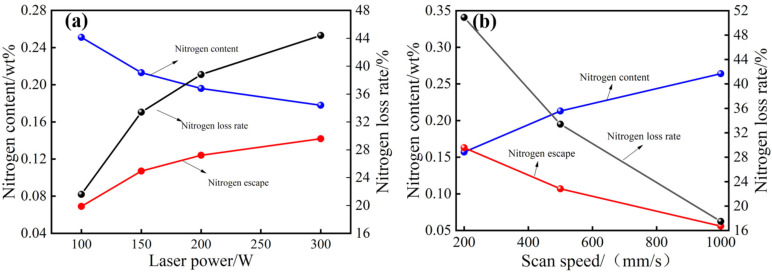
Nitrogen emission of SLM sample: (**a**) different laser power; (**b**) different scan speed.

The effects of the process parameters on the nitrogen content of the samples were analysed. The main parameter values that were tested were: laser powers of 100, 150, 200, and 300 W; scanning speeds of 200, 500, and 1000 mm/s; protective gas of nitrogen; and environmental pressure of 0.003 MPa. The results are shown in Figure 3.

Figure 3a shows that at a laser power of 100 W, the nitrogen content in the sample is 0.251 wt.%, the nitrogen escape amount is 0.069 wt.%, and the nitrogen loss rate is 21.6%. At a laser power of 300 W, the nitrogen content is 0.178 wt.%, and the nitrogen loss rate is 44.4%. With an increase in laser power, the nitrogen content decreases by 0.073 wt.%, and the nitrogen loss rate increases by 22.8%. Figure 3b shows that at the scanning speed of 200 mm/s, the nitrogen content in the sample is 0.157 wt.%, the nitrogen emission is 0.163 wt.%, and the nitrogen loss rate is 50.9%. At the scanning speed of 1000 mm/s, the nitrogen content in the sample is 0.264 wt.%, the nitrogen emission is 0.056 wt.%, and the nitrogen loss rate is 17.5%. With an increase in the scanning speed, the nitrogen emissions decrease by 0.107 wt.%, and the loss rate decreases by 33.4%.

The loss of nitrogen in the sample increases with the increase in laser power, and the increasing trend is clear in the laser power range of 100–300 W. With an increase in laser power of 50 W, the nitrogen loss rate increases by 11.8%. In the range of 150–300 W, the increasing trend gradually slows: with an increase in laser power of 150 W, the nitrogen loss rate increases by 11%. However, the loss of nitrogen in the sample decreases with an increase in scanning speed. In the range of 200–500 mm/s, the nitrogen loss rate decreases significantly: an increase in the scanning speed of 300 mm/s corresponds to a decrease in the nitrogen loss rate of 17.5%. In the range of 500–1000 mm/s, the amount of nitrogen escaped decreases slowly: with an increase in the scanning speed of 500 mm/s, the nitrogen loss rate decreases by 15.9%.

Under low nitrogen partial pressure (0.003 MPa), the nitrogen content in the molten pool formed by SLM exceeded the saturation solubility; thus, nitrogen escape occurred. At constant scanning speed, energy absorbed by the powder increases with the increase in laser power, which in its turn, increases the time during which the melting state is maintained and even causes the powder to appear over the molten pool and directly form a metal vapour plume. Higher laser energy impacts the molten pool and promotes the splashing of the solution in the molten pool, which promotes the discharge of nitrogen bubbles in the molten pool and aggravates the nitrogen escape problem. At a constant laser power, with the increase in the scanning speed, the energy absorbed by the powder per unit time decreases, the powder does not reach the over-melting state, and individual powder particles even remain in the unmelted state; thus, the amount of nitrogen escape decreases. In addition, with the increase in scanning speed, the action time of the laser becomes shorter, and the ‘disturbance’ effect of the laser on the molten pool decreases, which is not conducive to the rise of nitrogen bubbles in the molten pool and inhibits the escape of nitrogen during SLM.

**Figure 4:**
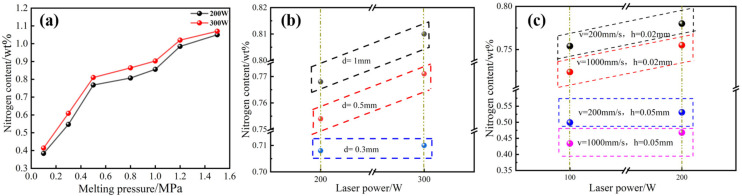
Analysis of nitrogen content in SLM samples with: (**a**) different cavity pressures; (**b**) different layer thicknesses; (**c**) different scanning intervals.

HPSLM of high-nitrogen stainless steel was performed at high laser powers (200 and 300 W) and a low scanning speed of 200 mm/s and different cavity pressures were considered. The nitrogen contents of the samples formed at different pressures are shown in Figure 4a. Figure 4b shows the nitrogen content of samples with different layer thicknesses, and Figure 4c shows the nitrogen content of samples with different scanning intervals.

Figure 4a shows that the nitrogen content of the HPSLM high-nitrogen stainless steel sample is in the range of 0.384–1.05 wt.%, and the nitrogen content of the sample is the lowest at a melting pressure (ΔP) of 0.1 MPa. At a ΔP of 1.5 MPa, the nitrogen content of the sample is the highest (1.05 wt.%). In the range of 0.1–0.5 MPa, the nitrogen content increases significantly. In the range of 0.5–1.5 MPa, the nitrogen content increases slowly. The nitrogen content in the sample increased with an increase in the cavity pressure. On the one hand, the increase in cavity pressure increases the partial pressure of nitrogen in the SLM melting and solidification, and [N] in the molten pool does not reach saturation. A large amount of nitrogen diffused into the micro-molten pool under the action of the laser during melting, and the amount of nitriding increased. On the other hand, after increasing the cavity pressure, the surface acting pressure of the molten pool increased, and nitrogen escape was inhibited. The experimental results show that the melting pressure in SLM can promote the infiltration of nitrogen and inhibit the escape of nitrogen, which is consistent with theoretical calculations.

Figure 4b shows that samples with different powder layer thicknesses have different degrees of nitriding. The amount of nitriding is proportional to the thickness of the powder layer. The nitrogen content of the samples with different thicknesses of the powder layer is in the range of 0.708 to 0.768 wt.%. At high nitrogen partial pressures, no nitrogen escape was observed. The powder bed absorbed the laser energy to form a molten pool. The heat transfer inside the molten pool was mainly conduction. The thermal conductivity of the powder layer was lower than that of the substrate. When the powder layer was thicker, heat conduction was lower. The molten pool extends and melts the powder to form a large-scale molten pool, and the gas–liquid nitriding interface becomes larger, which provides good dynamic conditions for SLM nitriding.

Figure 4c shows that the nitrogen content in the samples with different scanning intervals is different, and the amount of nitriding is inversely proportional to the scanning interval. The main reason for this is that a sample of the same size is formed. The weld channel of the sample with a low scanning interval is larger than that of the sample with a high scanning interval, resulting in a higher number of molten pools in the sample produced at a low scanning speed compared to that in the sample produced at a high scanning interval. Under high pressure, the increase in the number of molten pools strengthened the nitriding behaviour and nitrogen content of the sample.

### 3.2. Phase Composition and Nitrogen Distribution

**Figure 5:**
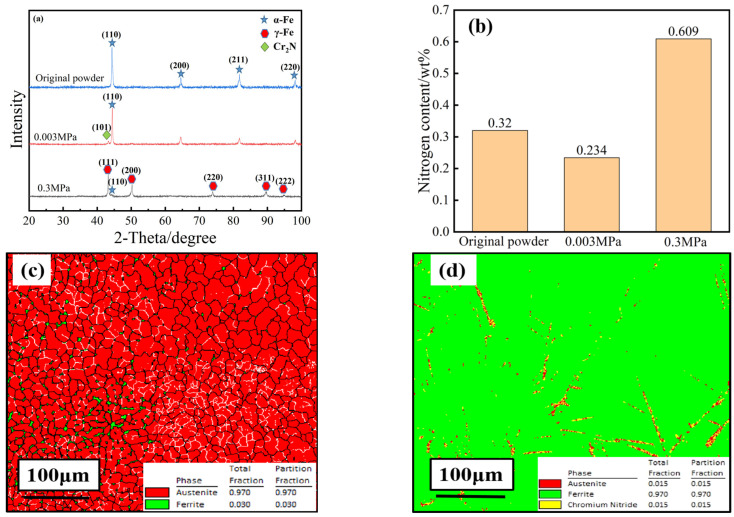
Phase composition of the samples with different nitrogen contents: (**a**) XRD results for samples; (**b**) nitrogen content of the samples; (**c**) EBSD results for the 0.3 MPa sample; (**d**) EBSD results for the 0.003 MPa sample.

Nitrogen escape and infiltration occur during SLM of high-nitrogen stainless steels. During rapid solidification, unconventional phase transformation behaviour may occur. The phase composition analysis of samples with different nitrogen contents is helpful to explore the phase transformation behaviour of the SLM rapid solidification and analyse the effect of nitrogen content on the phase composition of the samples. The original powder, a 0.3 MPa HPSLM sample, and a 0.003 MPa SLM sample were characterised by XRD, and the test results are shown in Figure 5.

Figure 5a shows that the original high-nitrogen stainless steel powder consists of a single α-ferrite phase, corresponding to the (110), (200), (211), and (220) crystal planes. The main phase of the 0.003 MPa sample does not change, and the α-ferrite phase is maintained, which is consistent with the electron backscatter diffraction (EBSD) results for the 0.003 MPa sample in Figure 5d. However, oriented precipitates appear near the (110) orientation peak. Combined with the EBSD results, this suggests that the precipitates are nitrides, and the intensities of each diffraction peak of the α-ferrite phase are slightly weakened.

The main phase of the 0.3 MPa sample changed from the δ-ferrite phase to the γ-austenite phase, and there is a small amount of residual δ-ferrite, which is consistent with the EBSD results for the 0.3 MPa sample in Figure 5c. The γ-austenite phase corresponds to the (111), (200), (220), (311), and (222) crystal planes and the residual δ-ferrite corresponds to the (110) crystal plane. The γ-austenite phase diffraction peak has a small angle shift, which is attributed to the lattice distortion caused by the change in nitrogen content during HPSLM.

**Figure 6:**
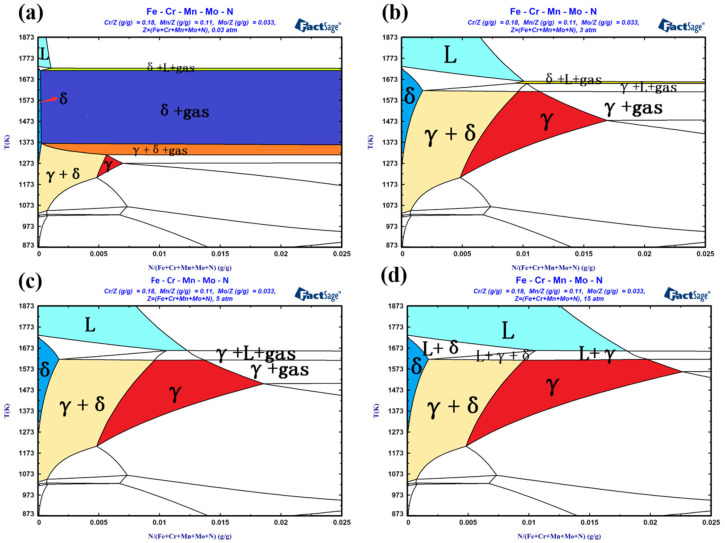
Fe-N binary phase diagram under different melting pressure: (**a**) 0.003 MPa; (**b**) 0.3 MPa; (**c**) 0.5 MPa; (**d**) 1.5 Mpa.

**Figure 7:**
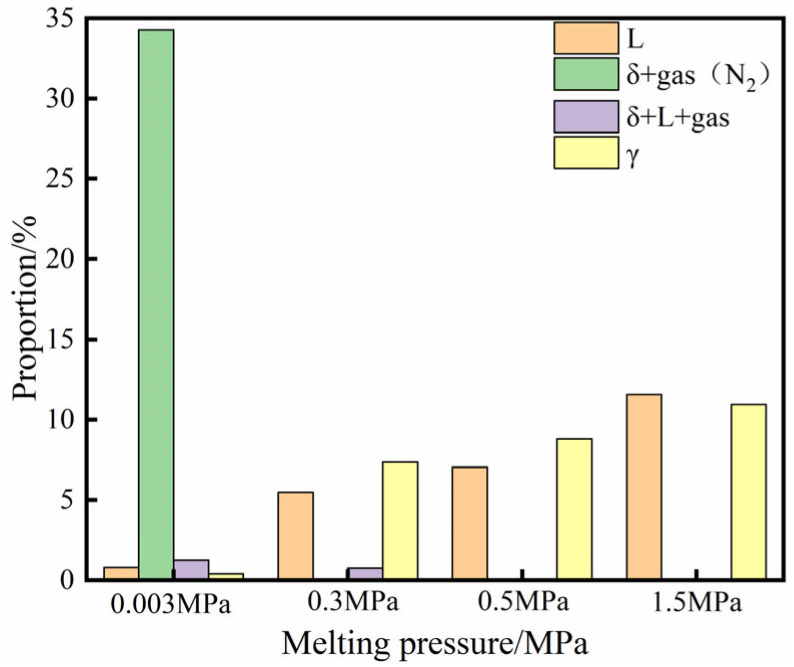
Proportion of each phase zone under different melting pressure.

To further analyse the phase transformation behaviour of high-nitrogen stainless steel powder obtained through SLM, the Fe–N binary phase diagram of the melt at different pressures is established, as shown in Figure 6. The effective area of the phase region is introduced, and the expression of the effective area ratio, N, of each phase region is given by Formula 1. The change in the proportion of each phase region under different pressures is calculated by applying the software grid method, as shown in Figure 7.

N = S_n_/S (1)

In Formula (1), N is the effective area ratio of the phase region, S_n_ is the area of the liquid phase, liquid phase + gas [N_2_], or γ-austenite phase (mm^2^), and S is the total area of the phase diagram (mm^2^).

A comparison and analysis of the Fe–N binary phase diagram and phase zone grid calculation results under different melting pressures revealed that the liquid phase area accounts for approximately 0.8% at a melting pressure of 0.003 MPa. With an increase in the melting pressure, the liquid phase area moves to the right and the proportion of the liquid phase increases. At a melting pressure of 1.5 MPa, the liquid phase area accounts for approximately 11.55%, indicating that the solubility of nitrogen in the melt increases with the increase in melting pressure.

Thus, the EBSD, XRD, and Fe–N binary phase diagrams at different melting pressures suggest that the solidification phase transformation of the HPSLM high-nitrogen stainless steel is as follows: liquid phase → δ-ferrite phase + liquid phase → liquid phase + δ-ferrite phase + γ-austenite phase → δ-ferrite phase + γ-austenite phase.

The solidification phase transformation of SLM high-nitrogen stainless steel is as follows: liquid + gas (N_2_) → δ-ferrite + liquid + gas → δ-ferrite + gas → δ-ferrite + γ-austenite + gas → δ-ferrite + γ-austenite. During SLM of high nitrogen stainless steel, limited by the low nitrogen partial pressure (0.003 MPa) of the forming chamber, nitrogen escape occurs in the initial liquid phase + gas (N_2_) and experiences a wide range of δ-ferrite phase + gas (N_2_), which aggravates the nitrogen escape, resulting in low nitrogen content in the solidification zone, which is not conducive to the formation, expansion, and stability of the γ-austenite phase, resulting in a small part of the solidification zone of γ-austenite phase. When HPSLM was performed, the limitation of low nitrogen partial pressure in the forming bin was removed. Under high nitrogen partial pressure, the solubility of nitrogen in the melt increased, and the nitrogen content of the solidification region increased, which was beneficial to the formation, expansion, and stability of the γ-austenite phase. The volume fraction of the γ-austenite phase increased with an increase in nitrogen content.

**Figure 8:**
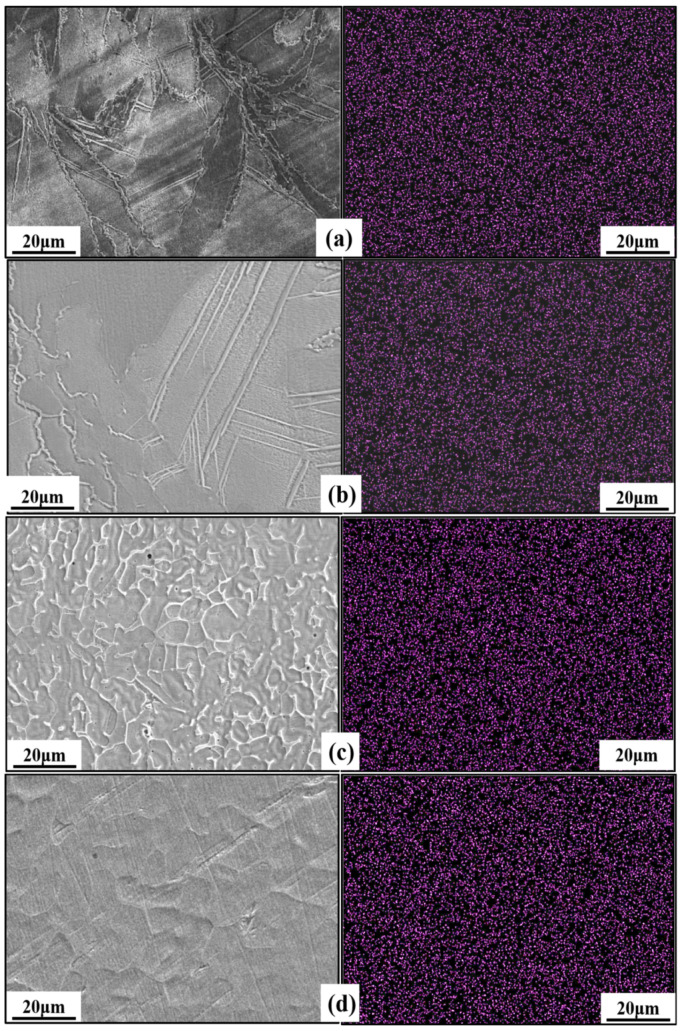
Micromorphology and nitrogen distribution in the samples formed using different SLM methods: (**a**) SLM overlay layers; (**b**) SLM scan layers; (**c**) X-Z cross-sections of the HPSLM specimens; (**d**) X-Y cross-sections of the HPSLM specimens.

To explore whether the nitrogen distribution in the high-nitrogen stainless steel samples formed by using SLM was uniform, scanning electron microscopy of the surface of the SLM and HPSLM samples was performed, and the surface scanning results are shown in Figure 8. A comparison of the scanning results of different cross-sections of SLM and HPSLM specimens showed that nitrogen is evenly distributed in the overlay layer, scanning layer of SLM specimens, and the X–Y and X–Z cross-sections of the HPSLM specimens, and no obvious element segregation occurred. The scanning results of different cross-sections of different SLM samples indicated that the micro-pool superposition principle and rapid solidification characteristics of SLM can effectively inhibit element segregation.

### 3.3. Microstructure of High-Nitrogen Stainless Steel

**Figure 9:**
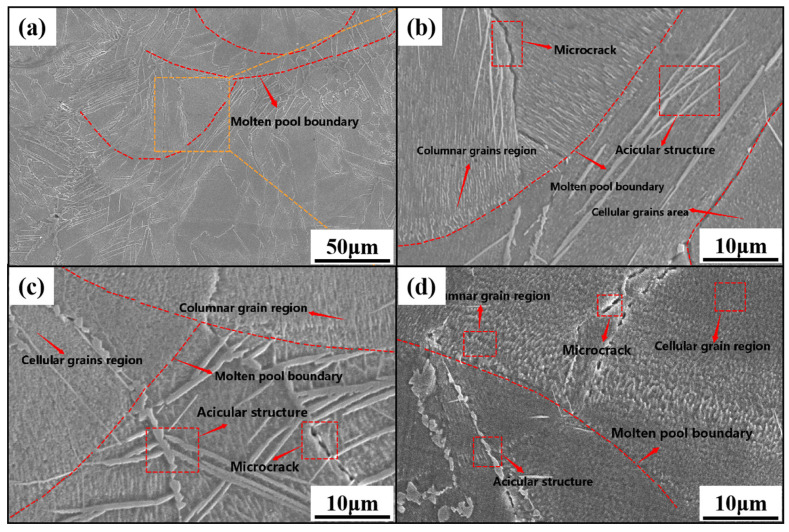
Microstructure of formed block at different scanning speeds:(**a**,**b**) 1000 mm/s; (**c**) 500 mm/s; (**d**) 200 mm/s.

The microstructures of the samples obtained at different scanning speeds are shown in Figure 9. The microstructures of the samples at different scanning speeds consist mainly of columnar and cellular crystals and do not change significantly with the decrease in scanning speed. At the edge of the columnar crystal region, a needle-like structure was generated. As shown in Figure 9b–d, the needle-like structure morphology changes from strip to staggered to droplet type with the decrease in scanning speed. Cracks appear near the boundary of the molten pool, and with the decrease in scanning speed, the crack size decreases from 8 to 5 to 3 μm. Two columnar crystals with different growth directions formed on both sides of the crack, as shown in Figure 9b–d. The columnar crystal region is at the edge of the molten pool, and the columnar crystals grew toward the centre along the edge. With the decrease in the scanning speed, the columnar crystal gradually coarsened, extending from the edge of the molten pool to the inside, and transforming from the columnar to the cellular crystal region. The cellular crystal size is approximately 1 μm.

This behaviour arises because, in the SLM forming process, the formed molten pool morphology is approximately hemispherical, and there is a large temperature gradient perpendicular to the laser direction at the edge of the molten pool. The grains grew along the direction parallel to the temperature gradient direction, forming a columnar crystal zone that preferentially grew along the heat flow direction from the molten pool boundary to the centre of the molten pool. However, the temperature gradient inside the molten pool was small, and the heat source distribution was uniform. After rapid solidification, a cellular crystal zone was formed. The crack at the boundary of the molten pool is mainly attributed to the short time of the powder–laser interaction at high scanning speeds and incomplete melting of a part of the powder, which reduced the degree of metallurgical bonding. In addition, there was high internal thermal stress, resulting in internal crack defects. With a decrease in scanning speed, the powder melted completely, improved the degree of metallurgical bonding, and alleviated the expansion of cracks formed during stress release.

**Figure 10:**
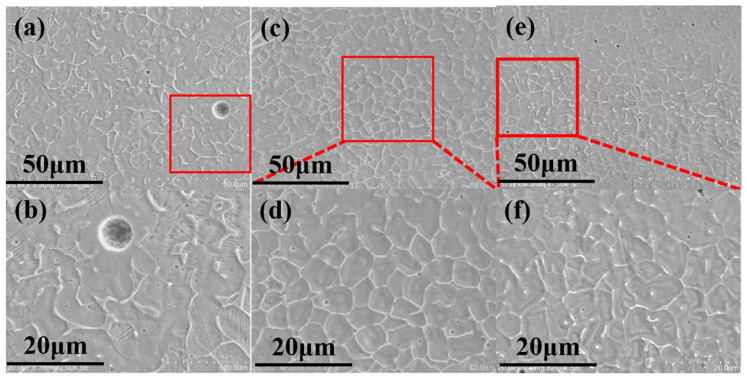
Micromorphology of samples under different cavity pressures: (**a**,**b**) 0.3 MPa; (**c**,**d**) 0.5 MPa; (**e**,**f**) 1.5 MPa.

The microstructures of the formed high nitrogen stainless steel under different chamber pressures are shown in Figure 10. Under such experimental conditions, the grain size decreases with the increase in the cavity pressure. In the range of 0.3–0.5 MPa, the grain size changes distinctly, whereas, in the range of 0.5–1.5 MPa, it changes slightly. Under the pressure in each chamber, the grain morphology is equiaxed.

### 3.4. Vickers Hardness of High-Nitrogen Stainless Steel

**Figure 11:**
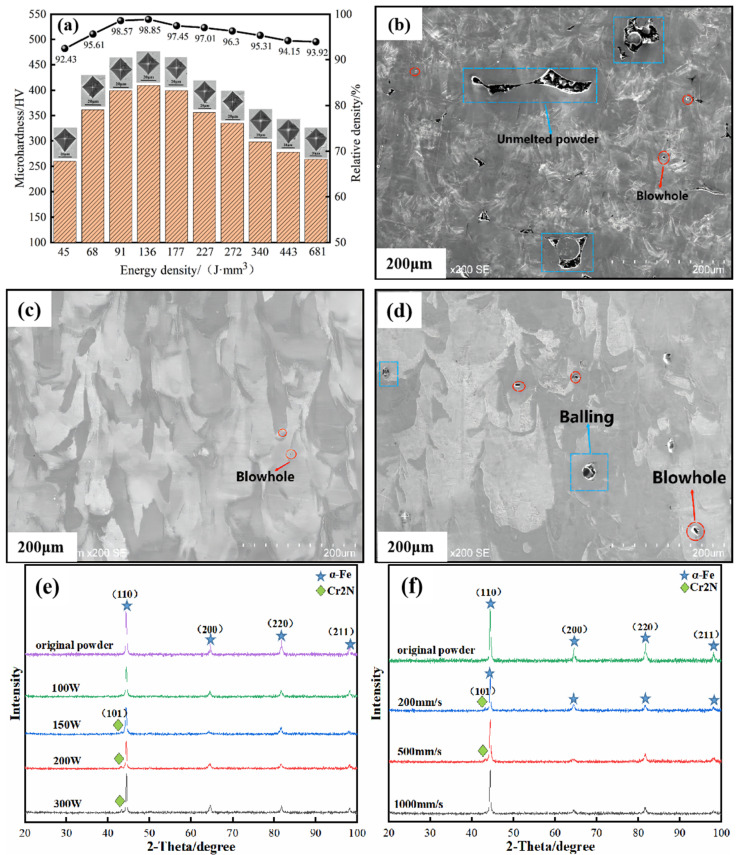
Microhardness distribution of the samples formed at different energy densities: (**a**) microhardness; (**b**) micromorphology of sample at 45 J/mm^3^; (**c**) micromorphology of sample at 136 J/mm^3^; (**d**) micromorphology of sample at 681 J/mm^3^; (**e**) XRD test at different scan speed; (**f**) XRD test at different laser power.

The microhardness and relative densities of the SLM high-nitrogen stainless steel samples are shown in Figure 11. It can be seen that the microhardness and relative densities under different laser energy densities are 260–409 HV and 92.43–98.85%y. At a laser energy density of 136.36 J/mm^3^, the microhardness and relative density reach the maximum—409 HV and 98.85%, respectively. The microhardness and relative density first increase and then decrease. As the laser energy density increases to 227 J/ mm^3^, the microhardness of the sample decreases by 54 HV, and the relative density decreases by 1.8%. The microhardness of the HPSLM samples with different cavity pressures is in the range of 245–281 HV. At a cavity pressure of 0.3 MPa, the microhardness is at a minimum (271 HV), while at a cavity pressure of 1.5 MPa, the microhardness reaches a maximum (345 HV). The increase in cavity pressure to 0.5 MPa does not result in a significant increase in microhardness (only a 10 HV increase). At the same time, a subsequent increase in the cavity pressure by 1.0 MPa results in a significant increase in microhardness (by 56 HV).

The microhardness of the SLM sample decreases with the increase in laser energy. This is attributed to a close relationship between the microhardness of the sample and the internal structure and phase composition of the sample. The above analysis and XRD test results for the SLM sample suggest that Cr_2_N precipitates in the sample with an increase in laser energy density. In addition, high laser energy density easily forms an ultra-high-temperature molten pool, and more metal vapour is formed. In addition, there were many internal defects in the sample formed under high laser energy density. Excessive pores and spheroidised particles worsened the internal microstructure of the sample, which affected its microhardness and relative density.

## 4. Conclusions

1. During the SLM of nitrogen-containing stainless steels, nitrogen emission was proportional to the laser power and inversely proportional to the scanning speed. The amount of nitriding during high-pressure SLM was proportional to the cavity pressure.
2. The main phase of the SLM sample did not change (was consistent with that of the original powder). However, after the increase in the pressure in the cavity, the phase changed from α-Fe to γ-Fe, and the peak position of the phase shifted due to the solid solution of nitrogen atoms. The nitrogen distribution in the samples formed by using different SLM methods was uniform without distinct element segregation.
3. At different scanning speeds, the internal structure of the sample consisted mainly of columnar and equiaxed crystals, accompanied by an acicular structure.
4. The microhardness and relative density of SLM samples first increased and then decreased with an increase in energy density and peaked at 136 J/mm^3^ (409 HV and 98.85%, respectively).

## Figures and Tables

**Table 1:** Chemical composition of nitrogen-containing stainless steel (wt.%).

Si	Cr	N	Mn	Mo	Ni	C	O	S	P	Fe
0.96	18.06	0.32	11.11	3.13	0.006	0.069	0.062	0.004	0.012	Bal

## Data Availability

No new data were created or analyzed in this study. Data sharing is not applicable to this article.
